# A systematic mapping of funders of maternal health intervention research 2000–2012

**DOI:** 10.1186/s12992-014-0072-x

**Published:** 2014-10-29

**Authors:** Katharine Footman, Matthew Chersich, Duane Blaauw, Oona MR Campbell, Ashar Dhana, Josephine Kavanagh, Mari Dumbaugh, Siphiwe Thwala, Leon Bijlmakers, Emily Vargas, Elinor Kern, Francisco Becerra, Loveday Penn-Kekana

**Affiliations:** Department of Infectious Disease Epidemiology, London School of Hygiene and Tropical Medicine, Keppel Street, London, WC1E 7HT UK; Centre for Health Policy, Faculty of Health Sciences, University of Witwatersrand, Johannesburg, 2000 South Africa; International Centre for Reproductive Health, Department of Obstetrics and Gynecology, Ghent University, De Pintelaan 185 UZP114, 9000 Gent, Belgium; Wits Reproductive Health and HIV research Unit, Faculty of Health Sciences, University of Witwatersrand, Johannesburg, 2000 South Africa; Department of Childhood, Families and Health, Institute of Education, 20 Bedford Way, London, WC1H 0AL UK; Independent Consultant, World Health Organization, Geneva, Switzerland; Radboud University Medical Center, Department of Epidemiology, Biostatistics and Health Technology Assessment (HEV), Nijmegen, The Netherlands; Centre for Health Systems Research, National Institute of Public Health, Colonia Santa María Ahuacatitlán, Cerrada Los Pinos y Caminera, C.P. 62100 Cuernavaca, México; Pan American Health Organization, 525 Twenty-third Street, N.W., Washington, D.C. 20037 USA

**Keywords:** Research, Funding, Maternal health, Priorities, Evidence, Research and development

## Abstract

**Background:**

The priorities of research funding bodies govern the research agenda, which has important implications for the provision of evidence to inform policy. This study examines the research funding landscape for maternal health interventions in low- and middle-income countries (LMICs).

**Methods:**

This review draws on a database of 2340 academic papers collected through a large-scale systematic mapping of research on maternal health interventions in LMICs published from 2000–2012. The names of funders acknowledged on each paper were extracted and categorised into groups. It was noted whether support took a specific form, such as staff fellowships or drugs. Variations between funder types across regions and topics of research were assessed.

**Results:**

Funding sources were only reported in 1572 (67%) of articles reviewed. A high number of different funders (685) were acknowledged, but only a few dominated funding of published research. Bilateral funders, national research agencies and private foundations were most prominent, while private companies were most commonly acknowledged for support ‘in kind’. The intervention topics and geographic regions of research funded by the various funder types had much in common, with HIV being the most common topic and sub-Saharan Africa being the most common region for all types of funder. Publication outputs rose substantially for several funder types over the period, with the largest increase among bilateral funders.

**Conclusions:**

A considerable number of organisations provide funding for maternal health research, but a handful account for most funding acknowledgements. Broadly speaking, these organisations address similar topics and regions. This suggests little coordination between funding agencies, risking duplication and neglect of some areas of maternal health research, and limiting the ability of organisations to develop the specialised skills required for systematically addressing a research topic. Greater transparency in reporting of funding is required, as the role of funders in the research process is often unclear.

**Electronic supplementary material:**

The online version of this article (doi:10.1186/s12992-014-0072-x) contains supplementary material, which is available to authorized users.

## Background

The role of evidence in policy making and program design has increased markedly in recent decades, alongside improvements in tools for collating evidence. Research funding, in large part, determines the types and amount of evidence generated, and thus the evidence base for informed decision-making. Other factors also influence what research is done, such as the availability of testable hypotheses, interventions or technologies; a predilection for quantifiable and statistically significant results; and the political importance of a topic. Nevertheless, the priorities of funders oftentimes dictate the research agenda, and thus govern decisions about what kind of research is supported, by whom and for what ends [1].

Research funding decisions and the efficiency of such funding has attracted much critique [2,3]. Imbalances in health research are well recognised [3,4]. An estimated 93% of the world’s burden of preventable mortality occurs in low- and middle-income countries (LMICs), yet only 5% of research addresses health problems in these countries [5]. Funding of health research is well short of global targets – high income country (HIC) governments spend under 0.01% of their GDP on Research and Development (R&D) for health in developing countries; and LMICs well below 2% of their health budgets on research (averaging 0.18% of GDP in lower-income countries) [3,5].

Clearly, factors such as disease burden and the availability of cost-effective interventions do not correlate with the amount of attention given to a health issue, with some high profile diseases receiving a disproportionately large amount of funding [6,7]. Rather than research funding agendas being evidence-based and transparent, support for an issue often depends on how the issue is understood and portrayed within the global health community [6]. The comparative advantage of a donor is also important [8], with, for example, the Bill and Melinda Gates Foundation initially prioritising research in new technologies over studies of methods of service delivery [4], linked to their comparative advantage in technology and innovation [8].

Examining funding for global health research and the actors involved in setting research funding priorities is difficult [9,10]. Development assistance tracking studies show that development assistance has progressively targeted countries with higher rates of maternal mortality [11], but these studies often exclude estimates of health research funding by organisations whose primary purpose is not development, such as national health research agencies, pharmaceutical companies, and some private foundations, such as the Wellcome Trust [12].

Rigorous assessment of the sources of research funding and the priorities of different funders will promote good research governance, and facilitate scrutiny of research funding distribution and donor coordination [2]. This study aims to document the research funding landscape in maternal health interventions in LMICs. It uses the funding acknowledgements of a large database of peer-reviewed publications to map: the actors acknowledged as funders of maternal health research; the priority topics and regions they are funding; and changes occurring between 2000 and 2012.

## Methods

The review draws on a database of maternal health literature developed by the MASCOT/WOTRO project, which is a large-scale systematic mapping of all maternal health interventions in LMICs published from 1 January 2000 to 31 August 2012. A systematic mapping of a broad body of literature on a topic differs from a systematic review, which addresses a single, clearly-defined, research question [13]. Systematic maps take into account all papers published on a topic, and seldom assess the quality of the research being mapped. This approach was considered appropriate for summarising the funding of the overall body of maternal health interventional research.

The MASCOT/WOTRO systematic mapping developed a sensitive search strategy, using both controlled vocabulary and free-text terms to identify studies on Medline (PubMED), which was adapted for subsequent searches of other electronic sources (Additional file 1). Seven databases were searched in total (Cumulative Index to Nursing and Allied Health Literature (CINAHL), Embase, LILACS, Medline, PopLINE, PsycINFO and Web of Knowledge).

To be included in the mapping, studies on maternal health had to address health system or health promotion interventions, community-based interventions, or single clinical interventions on four tracer conditions: haemorrhage, hypertension, HIV and other STIs, and malaria. The tracer conditions were selected as they constitute the two most common causes of direct maternal deaths (haemorrhage and hypertension), and as HIV and malaria are key causes of indirect maternal deaths in many settings [14]. We excluded articles on single-clinical interventions for non-tracer conditions, interventions related to fertility or infertility, and descriptions of coverage of routine services. The latter articles were excluded as the diversity of these studies made it very difficult to standardise data extraction across a large review team. The target population for interventions had to consist of a maternal health population (women in pregnancy, childbirth, or within two years postpartum), or men involved with a maternal health population (male involvement). General health system interventions were included provided that they reported on outcomes in a maternal health population. Outcomes could be both quantitative and qualitative, and all study designs collecting original data were included if they reported outcome(s) of an intervention. Systematic reviews were also included, but descriptive studies, narrative reviews, policy discussion papers, academic theses and books were excluded. Studies were included in the following languages: Arabic, English, French, Portuguese and Spanish.

**Figure 1:**
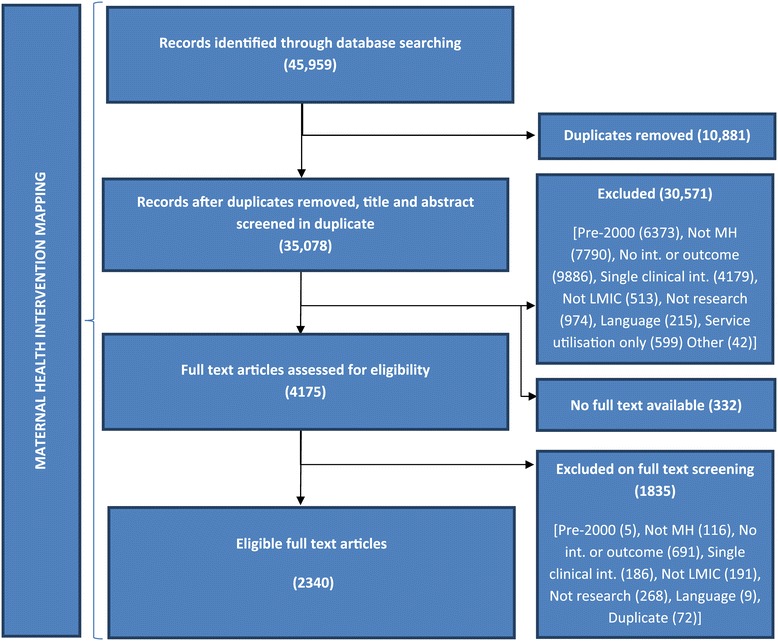
**Flow chart of overall systematic mapping.**

In total, 45,959 items were added to the online systematic review software EPPI-Reviewer 4 (eppi.ioe.ac.uk), of which 10,881 were duplicate items (Figure 1). The remaining records (35,078) were screened independently by two reviewers for relevance on their title and abstract. Differences between reviewers were resolved by a third reviewer. In total, 4507 records were marked for full text review. For 332 papers, full text documents could not be located. Of the remaining 4175 papers, 1835 (44.0%) were excluded after full text review. In total, 32,406 papers were excluded, mainly as they did not describe an intervention or outcome (10,577), or were single clinical interventions for a non-tracer condition (4476). Other reasons for exclusion were studies only providing data on utilisation of routine services (785), published before the year 2000 (6378), not on maternal health (7906), not in LMICs (704), not research (1242), and in an excluded language (224). The final mapping included 2340 papers.

Data extraction was carried out on full text papers in two stages. First, several variables were extracted from papers, including the country(ies) of study, country(ies) of the first author of the paper, study design, intervention topic, outcome data collected and intervention population. Details of the research funder were also extracted by directly copying the funding acknowledgements section of each paper or from any other part of an article where funders were mentioned. To locate information on funders, data extractors were instructed to search articles with the following terms: “fund”, “support”, “financ”, “acknowle”. In a second stage of data extraction, the individual names of every funder on each paper were extracted and classified into pre-specified categories. At this stage, it was noted if support was clearly stated to be ‘in kind’, in the form of pharmaceutical supplies, laboratory support, support for students, or staff fellowships and salary support. Where funding was said to be provided by a first organisation through a second organisation (e.g. this study was funded by funder X through a grant to organisation Y), only the first organisation (X) was taken to be the funder of the research. If funding was said to be provided by an organisation, and the paper also listed donors who had provided unrestricted support to the organisation, all listed funders were included.

Data were then transferred into Stata 13 (StataCorp LP, College Station, TX, USA) and coded. Funders were grouped into categories to facilitate analysis. We searched for existing categorisations of research funders, but as no agreed categorisation of research funders was located, we adapted a classification used in previous studies tracking official development assistance [12]. These categories were bilateral, multilateral, global health initiatives, private foundations, NGOs (international and national), private sector, and universities or research institutions. Additional categories for national research agencies (organisations such as the National Institutes of Health in the United States), LMIC governments and ‘other’ were noted as important during the review process and were included as funder types. Governments of LMICs were classified as bilateral if the funded study took place in another country, and were classified as national governments if the study took place in the same country as that of the government. Internet searches were done to gather information needed to accurately classify and categorise funders.

Descriptive analysis of the papers was carried out to assess the number of mentions of each category of funder, and the type of support specified. The number of papers funded by each category of funder was tabulated with the geographic region and intervention topic of papers. The major funders within each funder type were also identified, and the topics and countries of the major funders were then mapped. Analysis was also carried out to understand how funding patterns have changed over time.

## Results

### Who is funding maternal health intervention research?

Of the 2340 papers included in the review after full text screening, 1572 (67%) included details of their funding source in the funding acknowledgements. The remaining 768 papers either did not acknowledge their funding sources, or were unfunded. The number of funders acknowledged per paper ranged from one to fifteen (median 2; interquartile range 1–3).

A total of 685 different individual funders were mentioned 3266 times in the funding acknowledgements of the 1572 papers. Of the funders identified, 42% were acknowledged on more than one paper. The individual funders were categorised as bilateral (55 funders), multilateral (23), global health initiative (4), national research agency (47), university or research institute (169), international NGO (45), national NGO (25), private foundation (65), private sector (71), LMIC governments (40) and other (141). Funders included in the ‘other’ category included professional organisations (16), scholarships for individuals (22), hospitals (31), individuals (3), laboratories (2), regional medical offices (4), religious groups (4), national health services (2) and other non-profit organisations which could not be categorised as national/international NGOs or private foundations (20). Additionally, funders that could not be found on the internet (18) and funders that could not be classified as fitting into any of the above categories (19) were placed in the ‘other’ category.

The most common categories of funder were assessed in terms of how many times they appeared in funding acknowledgments as a proportion of all funder mentions (3266). This summed all instances where a funder was acknowledged, regardless of whether several funders of the same category were mentioned on the same paper. By this measure, the most common funders were bilateral (22% of funder mentions), national research agencies (21%) and private foundations (12%; Table 1).

**Table 1:** **Type of funding provided, by number of funder mentions**

**Funder type**	**No type**		**Staff**		**Student**		**Drugs**		**Laboratory**		**Total**	
	**N**	**%**	**N**	**%**	**N**	**%**	**N**	**%**	**N**	**%**	**N**	**%**
**Bilateral**	705	97%	15	2%	2	0%	4	1%	1	0%	727	22%
**Multilateral**	272	98%	2	1%	0	0%	0	0%	3	1%	277	8%
**Global Health Initiative**	35	95%	0	0%	0	0%	1	3%	1	3%	37	1%
**National Research Agencies**	652	93%	43	6%	3	0%	1	0%	0	0%	699	21%
**Universities**	316	88%	32	9%	9	3%	1	0%	1	0%	359	11%
**International NGO**	150	81%	13	7%	0	0%	19	10%	3	2%	185	6%
**National NGO**	31	91%	1	3%	0	0%	1	3%	1	3%	34	1%
**Private Foundations**	372	91%	32	8%	3	1%	1	0%	0	0%	408	12%
**Private Sector**	145	61%	21	9%	3	1%	57	24%	12	5%	238	7%
**National Government**	76	97%	1	1%	1	1%	0	0%	0	0%	78	2%
**Other**	179	80%	36	16%	6	3%	1	0%	2	1%	224	7%
**Total**	2933		196		27		86		24		3266	100%

### What type of funding is being provided?

The majority (90%) of funder mentions were not stated to be in any specific form, and were taken to be general financial support (Table 1). A tenth of funding mentions were for support in the form of drugs, laboratory support, staff fellowships or salary support, and financial support for students. Support in the form of staff funding was the most common type of support ‘in kind’ (6% of funder mentions), followed by donation of drugs (3%). While general financial support comprised more than 90% of funding for most funders, 39% of private sector funding was for support in kind, predominantly in the form of drugs (24%), staff funding (9%) or laboratory support (5%). Support in the form of staff funding was also more prominent among funders in the ‘other’ category (16%), followed by universities (9%) research agencies (8%) and international NGOs (7%).

### Which funders are dominating maternal health research funding?

**Figure 2:**
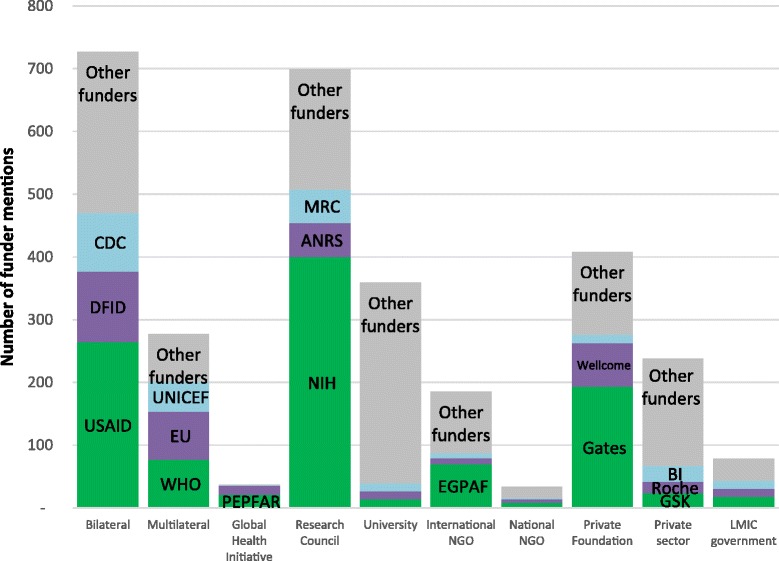
**Number of funder mentions attributed to the three largest funders in each funder type.** Abbreviations: DFID UK Department of International Development; CDC Centers for Disease Control; WHO World Health Organisation; EU European Union; PEPFAR US President’s Emergency Plan For AIDS Relief; ANRS Agence Nationale de Recherche sur le Sida; MRC Medical Research Council; EGPAF Elizabeth Glaser Pediatric AIDS Foundation; Gates Bill & Melinda Gates Foundation; Wellcome Trust; BI Boehringer Ingelheim; GSK GlaxosmithKline. *Unlabelled major funders:* Roll Back Malaria, Global Fund (Global health initiatives); L'Institut de recherche pour le développement (IRD), University of Copenhagen, University of North Carolina (Universities); Population Council, Medecins sans Frontieres (International NGOs); Health Foundation, ICDDR,B, Childbirth Connection (National NGOs); MacArthur Foundation (Private Foundations); Government of Thailand, Government of South Africa, Government of Brazil (National Governments).

Figure 2 shows the number of mentions of each funder type that are attributed to the three largest funders in each category. Several categories of funder, such as multilateral, bilateral, private foundations and national research agencies, were dominated by their three largest funders, with numerous other funders in each category making up a low proportion of funder mentions (3%-35%). The largest funders in other categories, such as the private sector, NGOs and universities, did not dominate their sectors, with funders other than the top three making up the highest proportion of overall funder mentions (53% to 89%).

As individual funders, the National Institute of Health (NIH) (12%), the US Agency for International Development (USAID) (8%) and the Bill and Melinda Gates Foundation (BMGF) (6%) made up the highest proportions of funder mentions. These funders together were acknowledged on 40% of all papers with a funder acknowledgement (and 22% of all papers, including those with no funder acknowledged). Other individual funders appearing on more than 5% of papers were the UK’s Department for International Development (DFID), the US Centers for Disease Control (CDC), the European Union (EU) and the World Health Organization (WHO).

### Where are funders focussing their research?

**Figure 3:**
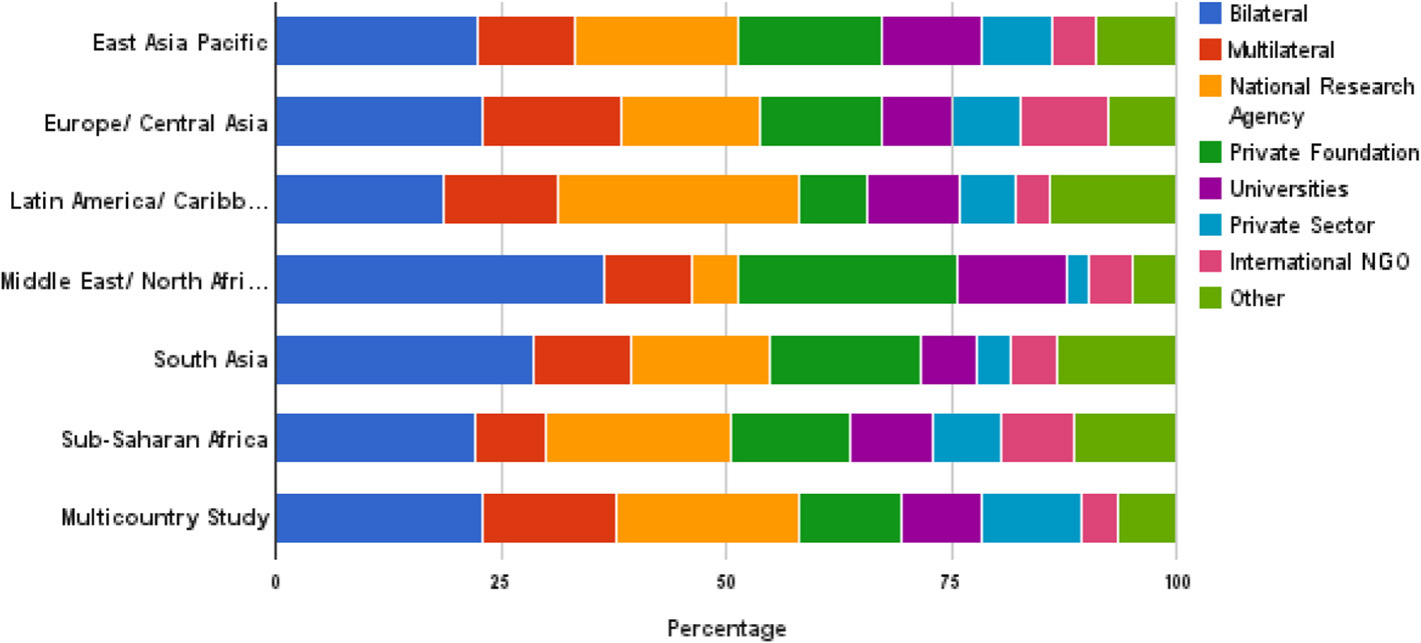
**The distribution of funder types by major regions.**

Figure 3 shows the funders that were most frequently acknowledged in each geographic region (including only studies with a funder acknowledged). Bilateral bodies were the most commonly acknowledged funder in every geographic region, ranging from 19% of papers on Latin America to 37% of papers on the Middle East/North Africa. National research agencies commonly funded papers in Latin America where they were acknowledged on 27% of papers, and they also made up 20% of multicounty studies and studies in sub-Saharan Africa. Private foundations played more of a role in the Middle East/North Africa region, where they funded 24% of papers.

**Figure 4:**
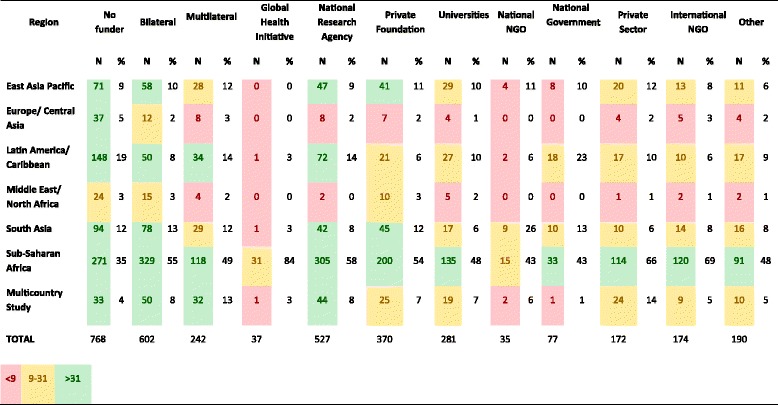
**Regions addressed by different funder types (as a % of all papers).**

Figure 4 shows the regions where each funder type most commonly funded research. For every funder, the most common geographic region funded was sub-Saharan Africa (43%-84% of their papers). Global Health Initiatives, in particular, have focused on sub-Saharan Africa (84% of the papers they funded). The second most common region for most funder types was East Asia/Pacific (0% to 12%), suggesting a limited geographical diversity across funder types.

### What types of intervention topics are being funded?

**Figure 5:**
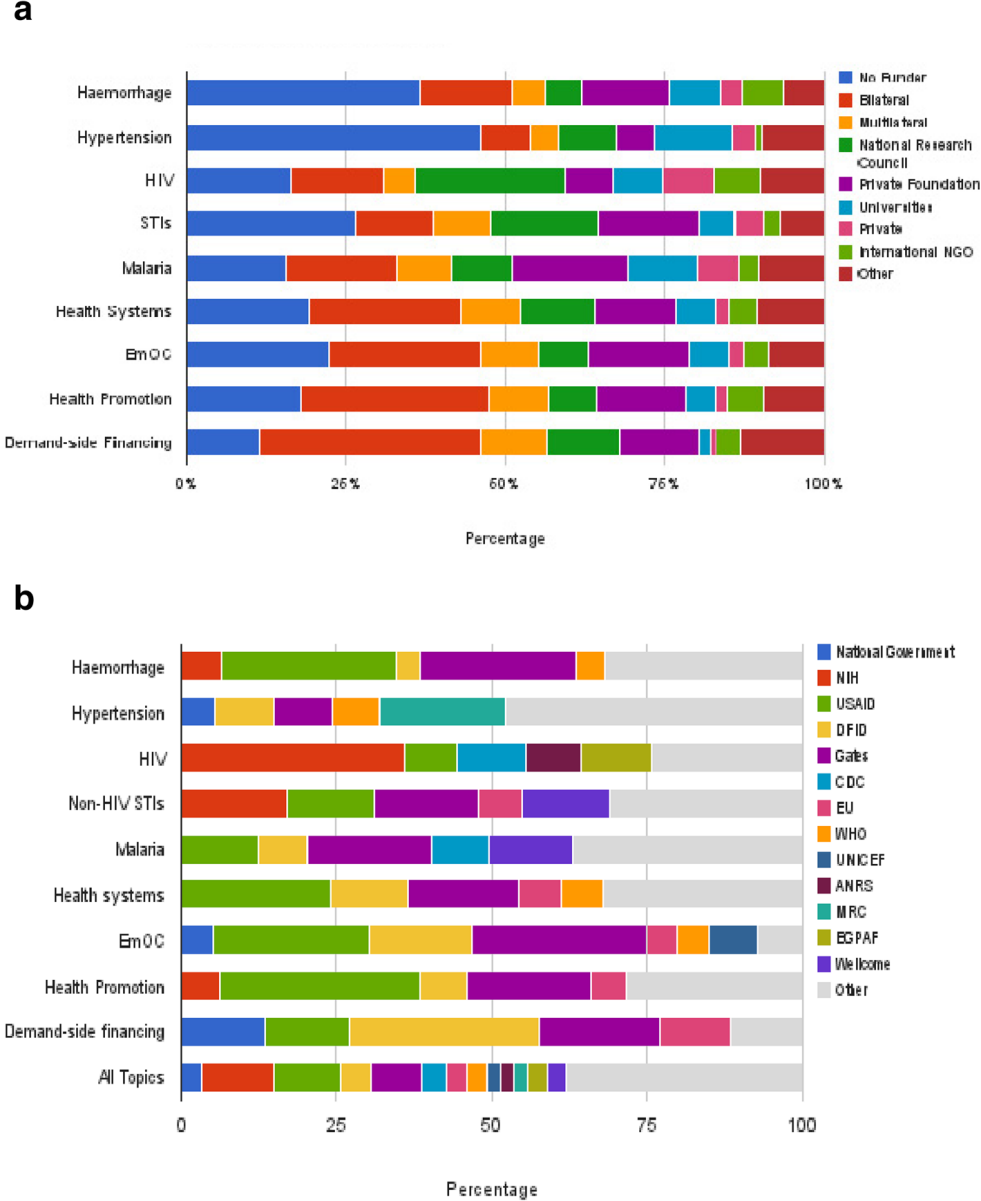
**The distribution of funder types by topic. a.** A stacked percentage bar chart showing the distribution of funder types for each topic. **b.** Five largest funders for each material health topic.

Figure 5a shows the funders that were most frequently acknowledged by papers on each intervention topic. The two main causes of maternal mortality – haemorrhage and hypertension – had a high proportion of papers without any funder (37% and 46% respectively). Studies on STIs other than HIV were also often without acknowledged external funding. National research agencies and private foundations seemed to play a greater role funding papers on HIV and STIs, while papers on malaria in pregnancy were not dominated by any particular funder type. Bilateral agencies played the greatest role in funding papers on health systems (24%), promotion (25%) and financing (35%).

**Figure 6:**
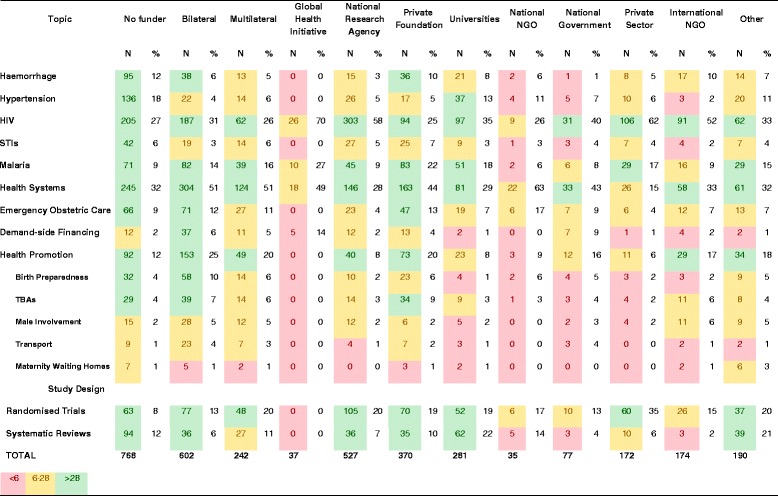
**Topics addressed by different funder types (as a % of total papers not funder mentions).**

Figure 5b shows the five largest funders acknowledged for each topic of maternal health. For topics such as HIV, there is clearly one dominant funder (NIH), whereas other topics such as emergency obstetric care, haemorrhage and health promotion have two or three dominant funders (USAID, Gates Foundation, DfID). Topics such as non-HIV STIs and hypertension are not clearly dominated by any one funder. This lack of a dominant funder may suggest that there is a broader range of funders with an interest in these topics. However, Figure 6 suggests that it might instead reflect the fact that there are no funder types prioritising these topics. Figure 6 shows the intervention topics that each funder type most commonly funded. For every funder type, the main focus was HIV (ranging from 25% to 70% of papers) and health systems interventions (ranging from 15% to 63% of papers). Topics such as haemorrhage, hypertension and STIs did not seem to be prioritised by any category of funder, ranging from 0 to 13% of each funder’s papers.

### How has funding changed over time?

**Figure 7:**
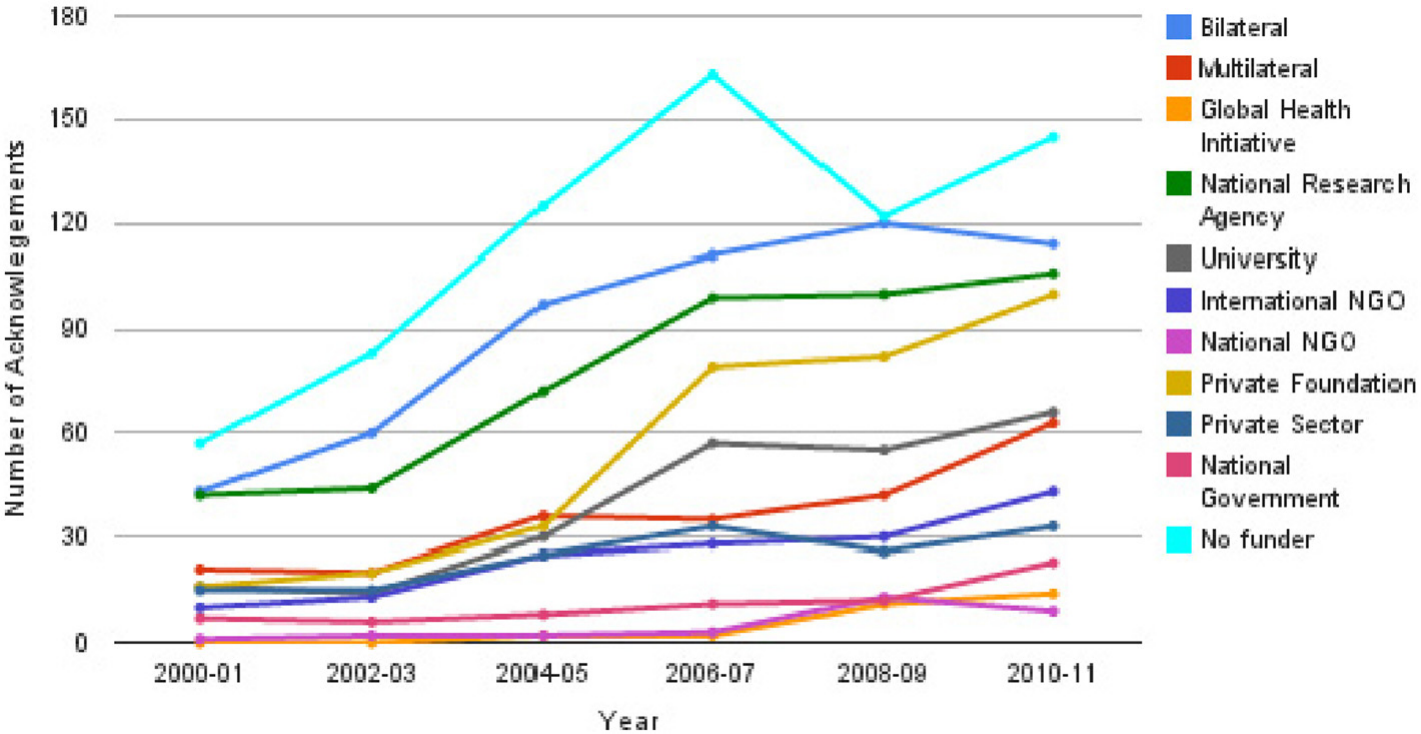
**Changes in the number of papers acknowledging each funder type between 2000–2011.**

Finally, we examined variations in the number of papers acknowledging each type of funder over the time period 2000 to 2011. Though the publication output for each funder type rose over time, different rates of increase were noted, and outputs plateaued for some funders (Figure 7). The rate of increase in outputs slowed markedly or flattened for bilateral, private sector, university and national research councils after 2006. Funding by private foundations rose rapidly after 2004, while multilateral funding increased at a relatively constant rate. The number of papers acknowledging national NGOs, local governments and global health initiatives remained fairly low throughout the time period.

## Discussion

Almost 700 funders were located in this systematic mapping of maternal health intervention research. A small fraction of these funders dominate published research, supporting assertions that relatively few donors identify maternal health as a key focus area [15]. Most prominent in our review are bilateral funders and national research agencies, while private foundations also support a sizable proportion of research outputs. Few studies were funded by national governments or NGOs suggesting that although funding of research by the public sector in LMICs has risen over time [14,16], it remains suboptimal.

Though tracking research funding amounts and outputs is difficult [17], funding allocations for maternal health research are disproportionate to the burden of disease [18,19]. There were about half a million publications on health in LMICs between 2002 and 2011 [17], which dwarfs the 35,078 publications we located on maternal health in a similar period (before applying exclusion criteria). These inequities in funding were also noted in the field of neglected disease, which received only about 1% of all Research and Development (R&D) investments in 2010 [17]. Funding for HIV research dominates other maternal health conditions, as occurs in neglected diseases (about 70% of R&D is on HIV, malaria and tuberculosis) [20]. Importantly the two main causes of maternal mortality – haemorrhage and hypertension – had a high proportion of papers without any funder, suggesting there has been inadequate support for these key areas of research.

Private sector funding of drug and laboratory support is several fold more common than in other funder categories. The plateau noted in industry funding of maternal research in recent years has occurred in all health topics, though this is the most difficult funding source to track [21]. Under-funding of pharmaceutical R&D for maternal health is a symptom of a market failure. While specific global initiatives have successfully overcome market failures in malaria, for example, there have been no similar global initiatives for maternal health. Globally, maternal health R&D is comparable to that for single conditions in high-income settings (for example, fewer maternal health drugs are in the pipeline than for Crohn’s disease) [18]. Boosting R&D for maternal health will require incentives for pharmaceutical investment and measures to convince governments, philanthropic agencies and other donors of what could be achieved.

Strengthening R&D is an important complementary strategy to health service provision for addressing the global burden of maternal diseases [18,19]. The WHO Commission on Macroeconomics and Health identified maternal and perinatal health as second only to infectious disease as a priority for global health research [22]. In spite of the research councils of both India and South Africa identifying maternal and perinatal health as priorities, overall R&D investment by both governments is small, ($55.2 M and $175.8 M, respectively) [19]. India, in particular, allocated <5% of its grants in reproductive health and nutrition to maternal or perinatal health [19]. Clearly, the high level of priority accorded to maternal health research in global, donor [8] and national policy documents has not translated into actual funding.

The study identified commonalities across funder types in the intervention topics and geographic regions covered, with HIV the topic most commonly addressed by each category of donor, and the majority of studies for all funders being set in sub-Saharan Africa. This echoes previous resource-tracking studies [12], and geographical gaps identified in research, such as in Latin America and the Middle East [15]. Having all key players addressing similar topics and geographical areas likely duplicates efforts and diffuses focus among funders and researchers. It also surely signals the absence of a long-term, carefully planned, progressive accumulation of knowledge on a topic, built upon specialised skills in the area. Having only a few major funders limits diversity of funding [23], such that, if a condition is accorded low priority by these funders, such as STIs other than HIV, there are few alternative funding sources, and little research is done in that area. An alternative approach is worth considering: the key funders each take responsibility for research on certain topics and regions, and develop specialised expertise both within their organisation and in researchers. Coordination of health research and development at a global level is a long-standing objective, with many initiatives in this area [17,24,25]. Coordination would, however, necessitate sharing of information between funders, and joint priority setting, planning and action [24].

The methods employed in this research have been used to complement other ways of tracking research funding and outputs [17]. However, the study has a number of limitations. Funding acknowledgements of published papers likely incur reporting biases, for example, by overestimating funders that have publication output requirements in their funding. Additionally, limiting the mapping to certain health system interventions and tracer conditions, may underestimate the role of funders that prioritise other conditions, or formative research such as needs assessments or descriptive cross-sectional studies. Similarly, we were only able to count the number of papers acknowledging a funder, not the amount of funding provided, or the impact of the research. As the unit of analysis was publications, not studies, multiple papers from one study were double counted. Vague language, such as ‘support’, sometimes used in funding acknowledgements may misclassify funders that provided organisational or management support, rather than financial support. Finally, we only have information on the stated funder, not the source of the funders’ resources. Agencies’ funding is complex and interlinked, and the funding bodies acknowledged may have received funding from another donor [12,26].

This study is therefore not intended to compare the various roles of funding bodies and their financial contributions, but to facilitate scrutiny of research governance by mapping out the various actors at play, and the research that they fund. Certain categories of funder were the most dominant in our analysis - bilateral donors, national research agencies and private foundations. It is not possible to say whether these types of funder are more or less likely to promote an evidence-informed research agenda, as this will largely depend on the individual organisation. Certain types of funder have more commonly come under criticism, such as private sector funders for their potential vested interests influencing study outcomes [27,28], and private foundations for their potential lack of accountability, links to private parent corporations and their setting of funding priorities according to personal interests [15,23,29,30]. However, regardless of category, every funder has its own agenda and may face its own perverse incentives. It is the governance of funders and the mechanisms in place to promote informed funding decisions that will decide their ability to consider evidence when setting research agendas.

Aside from their role in setting the agenda for research, funders also have the potential to influence the research process of the studies that they fund. This study found there was insufficient information to summarise the role of funders in most studies, one of the STROBE reporting recommendations [31]. Standardising reporting of the types of funding provided would promote transparency about funders’ contributions and a better understanding of their roles. Tracking the ways in which funding agendas are set (the extent to which evidence influences these decisions) and the presence of inter-funder coordination, might also promote accountability in research governance. Efficiency of funders’ resource use could be examined, by contrasting the scale of funding provided by different actors with the outputs mapped by our study.

## Conclusion

In conclusion, there is considerable diversity in the funding sources acknowledged by maternal health intervention research, though only a handful account for most publication outputs. Bilateral funders, national research agencies and private foundations were most often acknowledged, while the private sector commonly provides support ‘in kind’, often taking the form of pharmaceuticals. Despite the diversity of funding sources, there appears to be much similarity between the priority regions and topics of the major funders, suggesting that coordination between funders is poor.

## Additional file

Additional file 1:
**Annex 1: Search strategies.**
